# New synergistic combination therapy approaches with HDAC inhibitor quisinostat, cisplatin or PARP inhibitor talazoparib for urothelial carcinoma

**DOI:** 10.1111/jcmm.18342

**Published:** 2024-05-02

**Authors:** Sarah Meneceur, Caroline E. De Vos, Patrick Petzsch, Karl Köhrer, Günter Niegisch, Michèle J. Hoffmann

**Affiliations:** ^1^ Department of Urology, Medical Faculty and University Hospital Düsseldorf Heinrich Heine University Düsseldorf Düsseldorf Germany; ^2^ Center for Integrated Oncology (CIO) Düsseldorf CIO Aachen Bonn Köln Düsseldorf Düsseldorf Germany; ^3^ Genomics and Transcriptomics Laboratory (GTL), Biological and Medical Research Center (BMFZ) Medical Faculty and University Hospital Düsseldorf, Heinrich Heine University Düsseldorf Düsseldorf Germany

**Keywords:** bladder cancer, cisplatin, HDACi, quisinostat, talazoparib

## Abstract

Urothelial carcinoma (UC) urgently requires new therapeutic options. Histone deacetylases (HDAC) are frequently dysregulated in UC and constitute interesting targets for the development of alternative therapy options. Thus, we investigated the effect of the second generation HDAC inhibitor (HDACi) quisinostat in five UC cell lines (UCC) and two normal control cell lines in comparison to romidepsin, a well characterized HDACi which was previously shown to induce cell death and cell cycle arrest. In UCC, quisinostat led to cell cycle alterations, cell death induction and DNA damage, but was well tolerated by normal cells. Combinations of quisinostat with cisplatin or the PARP inhibitor talazoparib led to decrease in cell viability and significant synergistic effect in five UCCs and platinum‐resistant sublines allowing dose reduction. Further analyses in UM‐UC‐3 and J82 at low dose ratio revealed that the mechanisms included cell cycle disturbance, apoptosis induction and DNA damage. These combinations appeared to be well tolerated in normal cells. In conclusion, our results suggest new promising combination regimes for treatment of UC, also in the cisplatin‐resistant setting.

## 1 INTRODUCTION

Urothelial carcinoma (UC) contributing to more than 90% of bladder cancers is a frequent malignancy^1^ as the 10th most common newly diagnosed cancer worldwide.^2^ Treatment options for patients with UC depend on stage and localisation of the disease while patients with non‐muscle invasive disease receive local therapy. Patients with muscle invasive and metastatic disease undergo multimodal therapy including radical surgical resection, (chemo) radiotherapy and/or systematic treatment. Although the 5‐year overall survival for UC patients approximates 70%, patients with advanced disease present with an inferior prognosis resulting in a 5‐year overall survival of less than 20%.^3^ Standard systemic chemotherapy for bladder cancer relies on cisplatin based combination treatment. However, during the course of the disease, acquired and inherited resistance to treatment hampers the response rate significantly. Also, immune checkpoint inhibitors and antibody drug conjugates recently introduced into UC treatment algorithms improved outcomes only in a subgroup of patients,^4^, ^5^ underpinning the need for the development of new therapies.

TCGA studies with large UC patient tissue cohorts revealed that epigenetic regulators are particularly frequently mutated.^6^ Other epigenetic enzymes are aberrantly expressed in UC, indicating that epigenetic changes contribute to tumour progression^7^ and may be valuable therapeutic targets.

Histone deacetylases (HDAC) are epigenetic regulators catalysing the removal of acetyl groups on histones and non‐histone proteins; they are categorized in four classes. Due to their effect on chromatin compaction and gene transcription and on their various target proteins, HDACs are involved in different biological processes such as cell cycle, cell death, differentiation, DNA damage repair.^8^ Class I HDAC (HDAC1, 2, 3, 8) are of particular interest as they were found to be overexpressed in various cancer types, including UC, where they are associated with high tumour grade.^9^, ^10^, ^11^ We have previously shown that targeting class I HDAC with pharmalogical inhibitors (HDACi) (e.g. romidepsin) was highly potent compared to pan‐inhibition or inhibition of class II HDACs and had antineoplastic effect in UC cell (UCC) lines.^12^, ^13^ HDACi may induce DNA damage, quantifiable by the DNA double strand break marker γH2AX.^14^, ^15^, ^16^ We also reported earlier that rather HDAC1 and HDAC 2 are better suited targets than HDAC 8 and 3.^13^, ^17^ Since romidepsin targets mainly HDAC1 and 2 we had characterized its effect on UC cells extensively earlier.^16^

In this study, we characterized the impact of the second generation HDACi quisinostat on UC cells. We were interested to compare quisinostat with romidepsin, because quisinostat is a highly potent inhibitor of HDAC1. Differences in isoenzyme specificity may have different effects and may allow us to understand the functional role of class I HDACs in UC. In addition, quisinostat presents an improved pharmacodynamic profile compared to romidepsin^18^, ^19^ and antineoplastic effects in various cancer types (breast, liver, melanoma, medulloblastoma).^18^, ^19^, ^20^ Quisinostat has been investigated in six clinical studies for both haematological and solid malignancies (NCT02728492, NCT02948075, NCT00676728, NCT01486277, NCT00677105, NCT01464112).

Although HDACi appeared to be promising therapeutic options as single agent in bladder cancer in vitro^12^ and in clinical studies in haematological malignancies,^21^ clinical studies testing the effect of HDACi mocetinostat and vorinostat in patients with UC and other solid tumours showed elevated toxicity and modest efficiency.^22^, ^23^ To optimize treatment response to HDACi, potentially reduce dosage, and tackle resistance, combination therapies with other epigenetic regulators such as DNA methyltransferase inhibitors or bromodomain inhibitors and with chemotherapy (cisplatin, gemcitabine) represent an interesting approach. Combination therapies have indeed shown promising results in preclinical^24^, ^25^, ^26^, ^27^ and clinical studies.^28^, ^29^ Additionally, HDACi have been combined with PARP inhibitors targeting DNA repair, showing synergistic effect in various malignancies.^30^, ^31^, ^32^, ^33^ Their synergistic effect with HDACi and other epigenetic regulators has been attributed to the ability of the latter to downregulate genes involved in DNA damage repair (such as *BRCA*). However, PARP enzymes are involved in other biological processes (chromatin compaction regulation, transcription regulation), providing supplementary grounds for their combinations with epigenetic regulators.^34^, ^35^

In this study, we evaluated the effect of quisinostat on DNA damage, cell cycle and death in UCC as compared to romidepsin. Further, combinations with cisplatin and with the PARP inhibitor talazoparib were tested to enhance the efficiency of quisinostat in UCC and their cisplatin‐resistant sublines. Putative side effects of treatment were analysed using benign uroepithelial HBLAK cells and dermal fibroblasts. Benign cells tolerated treatment, particulary reduced dosage that could be applied due to synergistic effects of combined treatment.

## 2 MATERIALS AND METHODS

### 2.1 Cell culture

Different human UCCs were used to represent the heterogeneity of UC: VM‐CUB1, RT‐112, UM‐UC‐3, J82, SW‐1710 and T24. UCCs were obtained from the DSMZ (Braunschweig, Germany) and Dr. H.B. Grossmann HB (Houston, USA). Two benign cell lines were also used as control: HBLAK, a spontaneously immortalized normal human uroepithelial cell line (Cellntec Advanced Cell Systems)^36^ and VHF2 dermal fibroblasts.^37^ UCC and VHF2 were cultivated in DMEM (Dulbecco's Modified Eagle Medium, 4.5 g/L D‐glucose, L‐glutamine, Gibco, Life Technologies Limited) supplemented with 10% heat inactivated fœtal bovine serum (Bio & Sell). HBLAK was cultivated in CnT Prime epithelial cell culture medium (Cellntec Advanced Cell Systems) and to detach the cells, accutase (Sigma‐Aldrich) was used.

Previously generated cisplatin‐resistant sublines J82 LTT, T24 LTT and RT‐112 LTT were maintained in cisplatin supplemented medium as recently described.^38^ Phase contrast images of quisinostat or DMSO treated cells were taken with the Nikon Eclipse microscope (Nikon) and the NIS elements software.

The identity of the cells is regularly verified by STR (short tandem repeat) analysis, and cells were tested for mycoplasma contamination by PCR.

### 2.2 MTT assay

Cells were seeded in 96 well plates and treated with increasing concentrations of romidepsin (Selleckchem), quisinostat (Selleckchem), cisplatin (Neocorp, Hexal), talazoparib (Selleckchem), or combined treatment. Compounds were dissolved in DMSO (Sigma Aldrich). In line with data in literature, we had determined in previous work that robust effects by HDACi treatment effects can be observed after 72 h.^12^, ^13^ Thus, 72 h post‐treatment, the MTT (3‐(4,5‐dimethylthiazol2‐yl)‐2,5‐diphenyltetrazolium bromide) reagent was added (Sigma Aldrich), absorbance was measured at 595 and 750 nm with BioRad iMark reader (BioRad). Absorbance relative to the DMSO control was calculated (%) as measurement of metabolic activity as a surrogate for cell viability. Senolyticum venetoclax (Hycultec; in DMSO) was applied for 72 h at IC_50_ dosage (HBLAK 0.35 μM; J82 6.7 μM) after 72 h pre‐treatment with cell line‐specific low dose of quisinostat (J82 20 nM, HBLAK 7 nM).

### 2.3 Clonogenicity assay

Cells were treated for 3 days with compounds and reseeded at low density in 6 well plates in triplicates. After 10–15 days, colonies were fixed in methanol and stained with Giemsa.

### 2.4 Flow cytometry (FACS)

For cell cycle analysis, cells were resuspended in Nicolletti buffer (0.1% Triton X100, 0.1% sodium citrate) with 50 μg/mL propidium iodide (Sigma Aldrich, in PBS) and 100 μg/mL RNase A (100 mg/mL, Qiagen) and incubated at room temperature for 30 min. For annexin V staining, cells were resuspended in 1 × annexin binding buffer with annexin V‐FITC (Miltenyi Biotec), propidium iodide (Sigma Aldrich, in PBS) and RNase A (100 mg/mL, Qiagen) and incubated at room temperature for 15 min. Samples were then processed with the MACSQuant Analyser.

For the senescence staining, the Senescence Event Senescence Green Flow Cytometry assay kit (ThermoFischer Scientific) was used according to the manufacturer's instructions. The Viobility 405/452 fixable dye (Miltenyi Biotec) was added. Phase contrast images were taken with the Nikon Eclipse microscope (Nikon); the NIS elements software was used.

### 2.5 Caspase 3/7 assay

After 72 h of treatment in 6 wells plates, cells were detached and suspensions were placed in a 96 well plate in triplicates. An equal volume of the caspase3/7 glo reagent (Promega) was added and samples were incubated at room temperature protected from light for 1 h. In parallel, Cell Titer Glo reagent (Promega) was added to a second plate with the same samples and incubated for 10 min at room temperature in the dark to assess cell viability and normalize the results. Luminescence was measured with the Wallac Victor 1420 Multilabel counter (Wallac oy). Caspase 3/7 glo luminescence results were normalized to the Cell Titer Glo luminescence results.

### 2.6 Protein analyses

Histones were isolated by acid extraction^39^ as previously described. Proteins were extracted from cells with RIPA buffer (Cell Signaling Technology) suplemented with protease inhibitor cocktail (Sigma Aldrich) and phosphatase inhibitor cocktail 3 (Sigma), sonicated 3 times 10 s at 40% amplitude and centrifuged at 12,000*g* for 10 min at 4°C. Proteins and histones concentration was assessed by Pierce BCA protein assay kit (ThermoFischer).

For western blots, 20 μg of proteins or 2 μg of histones were denaturated in 4× rotiload at 95°C for 5 min and loaded on polyacrylamide gels. Nitrocellulose membranes were blocked in 5% milk TBS‐T. Primary antibodies: anti‐γH2AX (80312S, D7T2V, 1/1000, Cell Signalling), anti‐cleaved‐PARP (9544, D214, 1/500, Cell Signalling), anti‐PARP (9532, 46D11, 1/1000, Cell Signalling), anti‐α‐tubulin (T5168, clone B512, 1/50000, Sigma Aldrich). After washing with TBS‐T, membranes were incubated with secondary HRP‐coupled antibodies (polyclonal rabbit anti mouse immunoglobulin/HRP 1/2000, polyclonal goat anti‐rabbit Immunoglobulin/HRP1/1000, Dako).

### 2.7 Immunofluorescence stainings

Immunocytochemistry stainings for γH2AX and p53BP1 were performed as described perviously.^16^ Images were taken by means of Zeiss Axio Observer Z1 microscope using the 25× objective. Images were processed using ZEISS ZEN 3.9 software.

### 2.8 Apoptosis array

The proteome profiler array—human apoptosis array kit (R&D Systems, Inc.) was used. UM‐UC‐3 and J82 were treated for 24 h and for 72 h with IC_50_ dosage of quisinostat; DMSO treatment was used as control. Cells were detached and counted for a cell suspension concentration of 1 × 10^7^ cells/mL in lysis buffer. Protein concentration was assessed with the Pierce BCA protein assay (ThermoFisher Scientific) and 350 μg of proteins were used for the assay. Arrays and samples were subsequently processed according to the manufacturer's recommendation. For quantification, the intensity of the signal for each dot was assessed with the Image Lab Software (BioRad).

### 2.9 IL‐6 ELISA

Secreted IL‐6 was measured in cell culture supernatants using Lumit® IL‐6 (Human) Immunoassay (Promega) according to the manufacturer's instructions by means of Wallac Victor 1420 Multilabel counter (Wallac oy). Supernatants of quisinostat treated cells were collected 3 and 7 days after treatment. As a control, ionizing radiation was applied once and supernatants collected after 3 days. Cells were radiated once with 20 Gy (at 175 kV and 15 mA, for 20 min) using Gulmay RS225 x‐ray system (X‐Strahl).

### 2.10 Statistical analysis

Graphpad was used for data visualization and statistical analysis. IC_50_ values were determined by non‐linear regression analysis in GraphPad. *p*‐values were determined to evaluate significance of the differences between groups. The CompuSyn software was used to assess synergism between drugs.^40^ Fraction affected (Fa) was calculated from the percentage of viable cells (%) as 1−(%/100). Based on the combination index (CI), drugs were qualified as synergistic (CI <1), additive (CI = 1) or antagonistic (CI >1).

## 3 RESULTS

### 3.1 Quisinostat affects cell viability, cell cycle and death in the low nanomolar range

**FIGURE 1:**
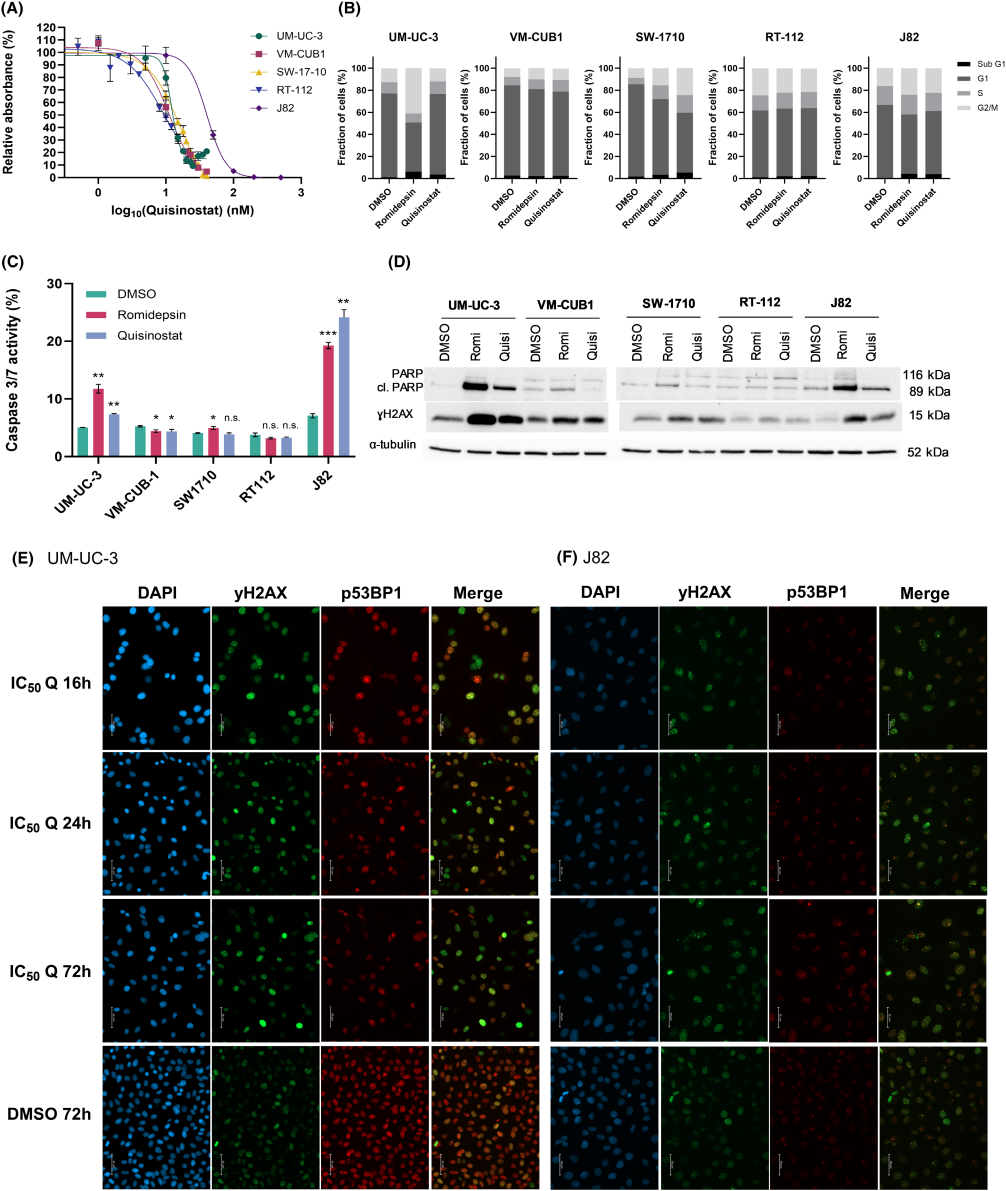
Quisinostat affects cell viability of UCC at low doses and has cell line dependent effects on DNA damage, cell death and cell cycle. (A) Non‐linear regression curves for determination of the IC_50_ values for quisinostat in UM‐UC‐3, VMCUB‐1, SW‐1710, RT‐112 and J82. Absorbance values for metabolic activity (MTT) relative to DMSO control (%) was calculated as surrogate for cell viability 72 h post‐treatment. (B) Bar graphs presenting the cell cycle distribution 72 h post‐treatment with HDACi romidepsin and quisinostat at IC_50_ values. Results of a representative experiment are shown, *n* = 3. (C) Caspase 3/7 activity in 5 UCCs 72 h post‐treatment with HDACi romidepsin and quisinostat at IC_50_ values. The results were normalized to the amount of viable cells measured with CellTiterGlo and are presented as percentage ±SD (standard deviation). *p*‐values are indicated as follows: *p < 0.05; **p < 0.005; ***p < 0.0005; n.s., non‐significant; *n* = 3. (D) Western blots showing the apoptopic marker cleaved PARP, total PARP and ɣH2AX following treatment with romidepsin and quisinostat after 72 h in 5 UCCs. α‐tubulin was used as loading control. The respective IC_50_ of the cell lines were used. Romi, romidepsin; Quisi, quisinostat. (E, F) Immunocytochemistry stainings of ɣH2AX (green fluorescence), p53BP1 (red fluorescence) and DAPI (blue fluorescence) or channel overlay (merge). Cells were treated with cell line dependent IC_50_ dosages of quisinostat (Q) (E, UM‐UC‐3 12.5 nM; F, J82 45 nM) and stained after 16, 24 and 72 h compared to DMSO. Images were taken with 25× objective, scale bar indicates 50 μm.

Dose response analysis of UCCs for quisinostat was performed (Figure 1A). Inhibitory concentration 50 (IC_50_) approximated 10 nM in VM‐CUB1, UM‐UC‐3, SW‐1710 and RT‐112 and was higher in J82, where it reached 40.9 nM. These values were higher than the IC_50_ obtained for romidepsin (1.7–5.8 nM) (Figure S1A,B). IC_50_ dosages respective to the cell lines led to an increase in the acetylated form of histone H3 (Figure S1C,D), confirming the effect of the HDACi at IC_50_ on histone acetylation.

Since HDACi are known to cause a G2/M cell cycle arrest in UCC, the effect of quisinostat on cell cycle was analysed (Figure 1B). In J82, SW‐1710 and to a lesser extent VM‐CUB1, the administration of romidepsin and quisinostat led to an increase in the proportion of G2/M cells. In UM‐UC‐3, while there were more cells in G2/M phase after treatment with romidepsin, quisinostat did not seem to affect cell cycle distribution. In RT‐112, both HDACi did not affect cell cycle distribution. Concerning cell death, romidepsin and quisinostat led to an increase in the proportion of necrotic and apoptopic cells in UM‐UC‐3, VM‐CUB1, J82, RT‐112 and SW‐1710 (Figure S1E). Caspase 3/7 activity was increased in UM‐UC‐3 and J82 after treatment with romidepsin and quisinostat, so was the apoptopic marker cleaved PARP in these cell lines (Figure 1C,D). Again in RT‐112, neither an increase in caspase 3/7 activity nor cleaved PARP was observed upon treatment with the HDACi suggesting this cell line to be rather unresponsive to quisinostat. To identify apoptopic proteins involved in the effect of quisinostat, an apoptosis array was done for UM‐UC‐3 and J82 after 24 and 72 h treatment with cell‐line specific IC_50_ values of quisinostat (Figure S2A–E). With the exception of the phosphorylated forms of p53 which were unexpectedly lower in both cell lines after treatment, other proteins displayed rather subtle changes. While Fas was only reduced after 72 h, HTRA2 was only increased after 72 h. Survivin, Claspin and XIAP were reduced after 24 h treatment and recovered after 72 h.

### 3.2 Quisinostat induces DNA damage

Since HDACi, particularly those targeting class I HDAC enzymes, can induce DNA damage, we tested the effect of quisinostat on DNA damage with the DNA double strand break (DSB) marker γH2AX in UCC. In all UCC, romidepsin and quisinostat led to an increase in γH2AX protein in western blot analyses (Figure 1D). In addition, we performed immunocytochemistry stainings for γH2AX and p53BP1 as established DNA double strand markers (DSB) after treatment with cell line dependent IC_50_ dosage of quisinostat for 16, 24 and 72 h. We chose the cell lines UM‐UC‐3 (Figure 1E) and J82 (Figure 1F) for stainings since these gave the most prominent increase in protein level in western blot analyses for γH2AX and cleaved PARP (Figure 1D). Images for different time points demonstrate that foci representing DSB were already detectable after 16 h and remained for 72 h after quisinostat treatment. DAPI staining further visualized enlarged nuclei of treated UC cells. Quisinostat could consequently be an appropriate combination partner with DNA damage inducing agents and DNA repair inhibitors.

### 3.3 Normal cells tolerate high doses of quisinostat and acquire a reversible senescence‐like phenotype

**FIGURE 2:**
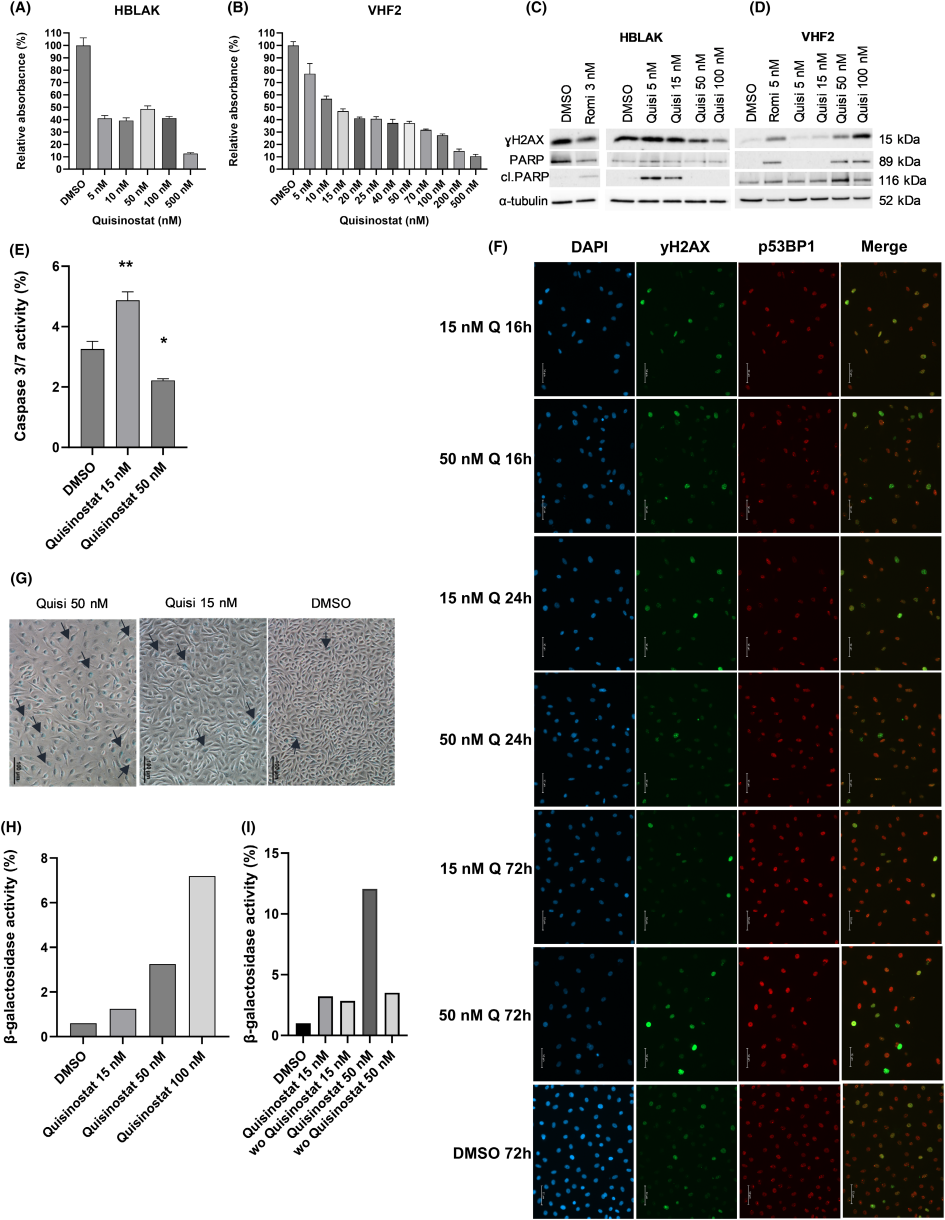
Normal cells tolerate high dosage of quisinostat and acquire a revertiblesenescence‐like phenotype. Bar graphs presenting metabolic activity as a surrogate for cell viability normalized to DMSO as relative absorbance (%) with increasing doses of quisinostat in the normal cells HBLAK (A) and fibroblasts VHF2 (B) 72 h post‐treatment. Western blots of the DNA double strand break marker ɣH2AX, the apoptopic marker cleaved PARP and total PARP with increasing doses of quisinostat in HBLAK (C) and VHF2 (D) 72 h post‐treatment, romidepsin was used for comparison. α‐tubulin was used as loading control. (E) Caspase 3/7 activity in HBLAK 72 h post‐treatment with quisinostat. The results were normalized to the amount of viable cells measured with CellTiterGlo and are presented as percentage ±SD (standard deviation). *p*‐values are indicated as follows: *p < 0.05; **p < 0.005; ***p < 0.0005; n.s., non‐significant; *n* = 3. (F) Immunocytochemistry stainings of ɣH2AX (green fluorescence), p53BP1 (red fluorescence) and DAPI (blue fluorescence) or channel overlay (merge). HBLAK cells were treated with either 15, 50 nM quisinostat (Q) or DMSO and stained after 16, 24 and 72 h. Images were taken with 25× objective, scale bar indicates 50 μm. (G) β‐galactosidase activity visualized with X‐gal staining 72 h after treatment with 15 and 50 nM quisinostat in HBLAK (scale bar 100 μm). (H) β‐galactosidase activity measured by FACS in HBLAK 72 h after treatment, *n* = 3. (I) β‐galactosidase activity measured by FACS in HBLAK 72 h after treatment compared to wash out (wo) after 24 h and measurement after 72 h, *n* = 3.

Quisinostat was applied to the benign uroepithelial cell line HBLAK and VHF2 fibroblasts to test its normal toxicity. Unlike romidepsin, for which the IC_50_ is below 10 nM in HBLAK and VHF2, quisinostat can be applied in much higher doses in both cell types (Figure 2A,B). Hence, in HBLAK, after a sharp decrease in metabolic activity measured by MTT assay at low dose of quisinostat, a plateau at 40% can be observed until 100 nM of quisinostat. In VHF2, metabolic activity decreases slowly between 5 and 500 nM, and the IC_50_ approximates 15 nM. Concurringly, in HBLAK, the apoptopic marker cleaved PARP can be observed upon treatment with 3 nM of romidepsin, 5 and 15 nM of quisinostat and is no longer observed with higher doses (Figure 2C). In VHF2, cleaved PARP appears after treatment with 5 nM of romidepsin, 50 and 100 nM of quisinostat, but not with 5 and 15 nM quisinostat (Figure 2D). The unexpected decrease in cleaved PARP observed with high doses of quisinostat in HBLAK was verified by measuring caspase 3/7 activity; it was observed that caspase 3/7 activity was also reduced after 50 nM quisinostat treatment of HBLAK cells (Figure 2E).

The protein level of DSB marker γH2AX was increased with HBLAK treated with 5 and 15 nM quisinostat, but decreased with higher doses (Figure 2C). In fibroblasts, it increased with the dose (Figure 2D). Additional immunocytochemistry stainings for HBLAK cells 16, 24 and 72 h after treatment with 15 or 50 nM quisinostat demonstrated that DSB were detectable to some extent after 16 h and remained after 72 h (Figure 2F). However, DNA damage levels seemed to be less pronounced compared to treated UCC (Figure 1E,F). Also HBLAK nuclei were less enlarged by treatment compared to treated UCC, but displayed lower DAPI intensity. Of note, in line with western blot results, staining of DMSO treated HBLAK revealed a quite high endogenous DNA damage level which may originate from their cellular origin. Corresponding bright‐field images show that treatment of HBLAK cells with 50 nM quisinostat did not increase significantly the number of dying cells, supporting our analyses of cleaved PARP and caspase activity (Figure S3).

Cell cycle distribution analysis indicated that low doses of quisinostat had little effect in HBLAK and VHF2, and 50 nM induced a cell cycle arrest in G2/M phase comparable to IC_50_ treatment with romidepsin (Figure S4A,B).

In HBLAK, we observed that cells presented morphological characteristics of a senescent‐like state with increasing doses of quisinostat (Figure S3A,B). In colony forming assays some cells seemed no longer to proliferate resulting in a moderate long‐term effect on proliferation (Figure S3C). β‐galactosidase staining revealed that high doses of quisinostat led to an increase in β‐galactosidase^+^ cells (Figure 2G), suggesting that quisinostat leads to senescence in HBLAK. To better quantify senescent cells, a FACS staining relying on the activity of β‐galactosidase was performed (Figure 2H) showing a dose dependent increase in the proportion of positively stained cells. To investigate whether benign HBLAK cells could recover from quisinostat treatment, we performed additional FACS experiments for activity of β‐galactosidase with a washout after 24 h of treatment. Interestingly, HBLAK treated with 50 nM clearly recovered after washout (Figure 2I), which became also visible by bright‐field microscopy (Figure S3B). Obviously, cells resumed cellular proliferation and grew as dense after 72 h as DMSO treated cells. Only few cells with senescent‐like morphology remained, particularly after treatment with 50 nM. In contrast, treated UCC displayed less prominent changes in cellular morphology (Figure S1F).

To further follow up on the senescence‐like state induced by quisinostat in benign HBLAK we used IL‐6 as another marker of senescence. IL‐6 levels secreted to media supernatant were increased 7 days after quisinostat treatment (Figure S3D) and comparable to ionizing radiation used as control. Also cellular morphology with enlargened flattened cells after radiation resembled the phenotype of quisinostat treated HBLAK.

Since senescent cells may be more sensitive to BCL‐2 inhibitors, which are used as senolytica, we compared response between HBLAK and J82 cells towards treatment with venetoclax after 72 h pre‐treatment with low dosage of quisinostat (IC_25_). Concurring with our other data on the quisinostat induced sensecent‐like state of HBLAK cells, they responded significantly stronger to the combination than J82 (Figure S3E). Taken together, our results point towards different mechanisms of response between UCC and benign HBLAK.

### 3.4 Quisinostat synergises with cisplatin and talazoparib in UCC

**FIGURE 3:**
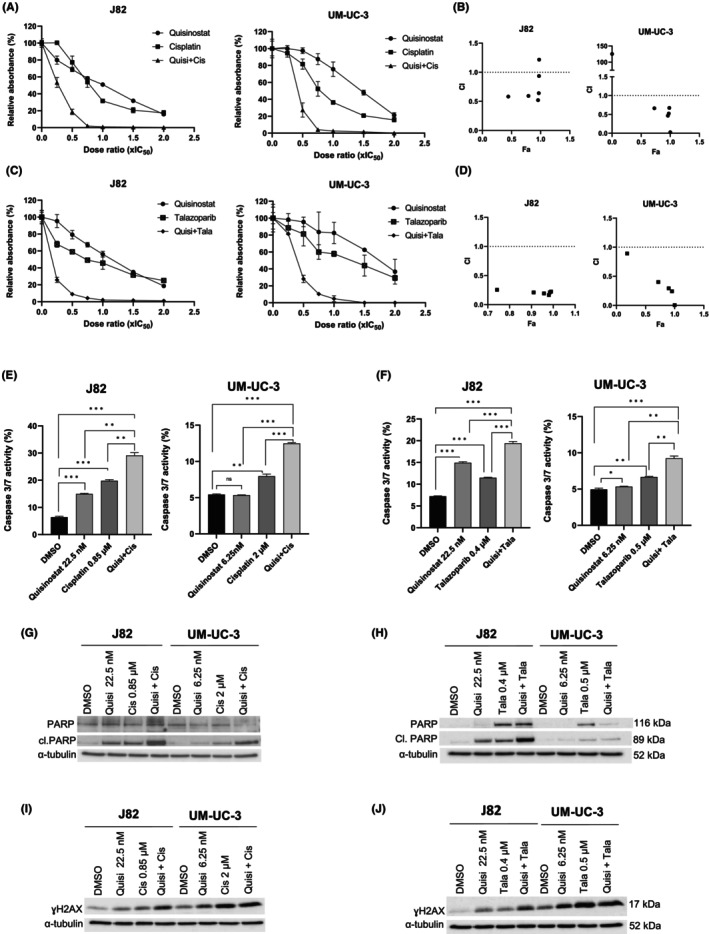
Quisinostat synergises with the DNA damage agent cisplatin and the PARP inhibitor talazoparib in UM‐UC‐3 and J82. (A, C) MTT assay curves of the single and combined treatments with cisplatin and talazoparib 72 h post‐treatment (± standard deviation SD), *n* = 4; (B, D) Chou Talalay analysis of the combinations quisinostat/cisplatin (C) and quisinostat/talazoparib (D), CI, Combination Index, Fa, Fraction affected, CI = 1: additive effect; CI >1: antagonistic effect; CI <1 synergistic effect. (E, F) Caspase 3/7 activity assay of the combined treatments at low dose ratios in J82 and UM‐UC‐3 72 h post‐treatment. The results were normalized to the amount of viable cells measured with CellTiterGlo and are presented as percentage ±SD (standard deviation). *p*‐values are indicated as follows: *p < 0.05; **p < 0.005; ***p < 0.0005; n.s. non‐significant; *n* = 3. Quisi, quisinostat; Cis, cisplatin; Tala, talazoparib; IC_50_, Inhibitory concentration 50; CI, combination index; Fa, fraction affected. (G, H) Western blots showing the apoptopic marker cleaved PARP (cl. PARP) and total PARP in UM‐UC‐3 and J82 treated with IC_25_ (0.5 × IC_50_) dosage of quisinostat, cisplatin, talazoparib, and combined treatment after 72 h. α‐tubulin was used as loading control. (I, J) Western blots showing the DNA damage marker γH2AX in UM‐UC‐3 and J82 treated with IC25 (0.5 × IC_50_) dosage of quisinostat, cisplatin, talazoparib, and combined treatment after 72 h. α‐tubulin was used as loading control.

Since quisinostat alone has moderate effect on UCC and was shown to induce an increase in the DSB marker γH2AX in UCC, combinations with cisplatin and the PARP inhibitor talazoparib were tested to optimize its therapeutic efficiency. Combining quisinostat with cisplatin (Figure 3A,B) or talazoparib (Figure 3C,D) led to a significant decrease in cellular metabolic activity compared to the single treatments at low dose ratio in J82 and UM‐UC‐3. The Chou Talalay analysis (Figure 3B,D) revealed that quisinostat synergises with cisplatin and talazoparib (CI <1) in both cell lines. Additional tests in other cell lines (VM‐CUB1, T24, RT‐112) indicated that both combinations led to a similar decrease and synergism (Figure S5). Thus, combined treatment with dosage below IC_50_ proved to be highly efficient. Since dose reduction will be further beneficial for normal toxicity, we performed further experiments with reduced dosage (0.5 × IC_50_).

**FIGURE 4:**
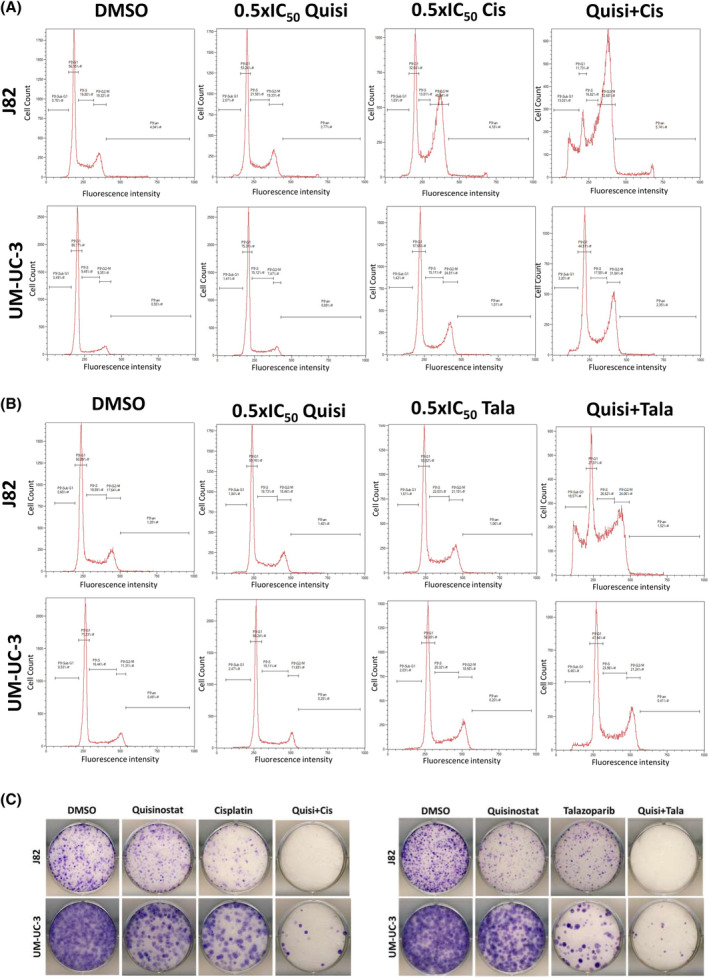
Combinations of quisinostat and cisplatin or talazoparib disrupt the cell cycle in UM‐UC3 and J82 at reduced dosage and affect long‐term proliferation. (A, B) Histograms showing the cell cycle distribution in UM‐UC‐3 and J82 following treatment with cell line‐specific IC_25_ dosage of quisinostat, cisplatin, talazoparib as single agents and combined treatment after 72 h, DMSO was used as control. Results of a representative experiment are shown, *n* = 3. Quisi, quisinostat; Cis, cisplatin; Tala, talazoparib; IC_50_, Inhibitory concentration 50. (C) Giemsa staining of the colony formation assay in UM‐UC‐3 and J82, after 72 h treatment with cell line‐specific IC_25_ dosage of quisinostat, cisplatin, talazoparib as single agents and as combined treatment, DMSO was used as control. Results of a representative experiment are shown, *n* = 3.

UM‐UC‐3 and J82 treated with both combined treatments quisinostat/cisplatin and quisinostat/talazoparib at low dose presented a higher caspase 3/7 activity (Figure 3E,F) and an increase in cleaved PARP (Figure 3G,H) compared to the single treatment, demonstrating an increase in caspase dependent apoptosis with combined treatments. γH2AX also increased with the combined treatments (Figure 3I,J). Annexin V stainings (Figure S6) also demonstrated an increase in the proportion of apoptopic cells with the combinations compared to single treatments. Both combinations resulted in an accumulation of cells in G2/M phase (Figure 4A,B). Additionally, a decrease in the long‐term proliferation was observed after treatment with the combinations in J82 and UM‐UC‐3 (Figure 4C).

### 3.5 Quisinostat synergises with cisplatin and talazoparib in cisplatin‐resistant sublines

As quisinostat was reported to restore chemosensitivity in various cancers, we investigated the effect of quisinostat in cisplatin‐resistant UCC sublines (referred to as LTT). Three LTT with different degrees of resistance to cisplatin were used: J82 LTT (moderately resistant, cisplatin IC_50_ = 26 μM); T24 LTT (resistant, cisplatin IC_50_ = 72 μM) and RT‐112 LTT (highly resistant, cisplatin IC_50_ = 200 μM). The IC_50_ of quisinostat for cisplatin‐resistant cell lines was determined: for J82, IC_50_ is similar to its parental counterpart (40 nM), while it is lower in the T24 cisplatin‐resistant subline compared to the parental T24 (7 nM in the LTT and 12.5 nM in the parental), and in RT‐112 (6 nM in the LTT and 10 nM in the parental cell line). These doses induced an increase in acetylated histone levels in LTT (Figure S7A,B).

**FIGURE 5:**
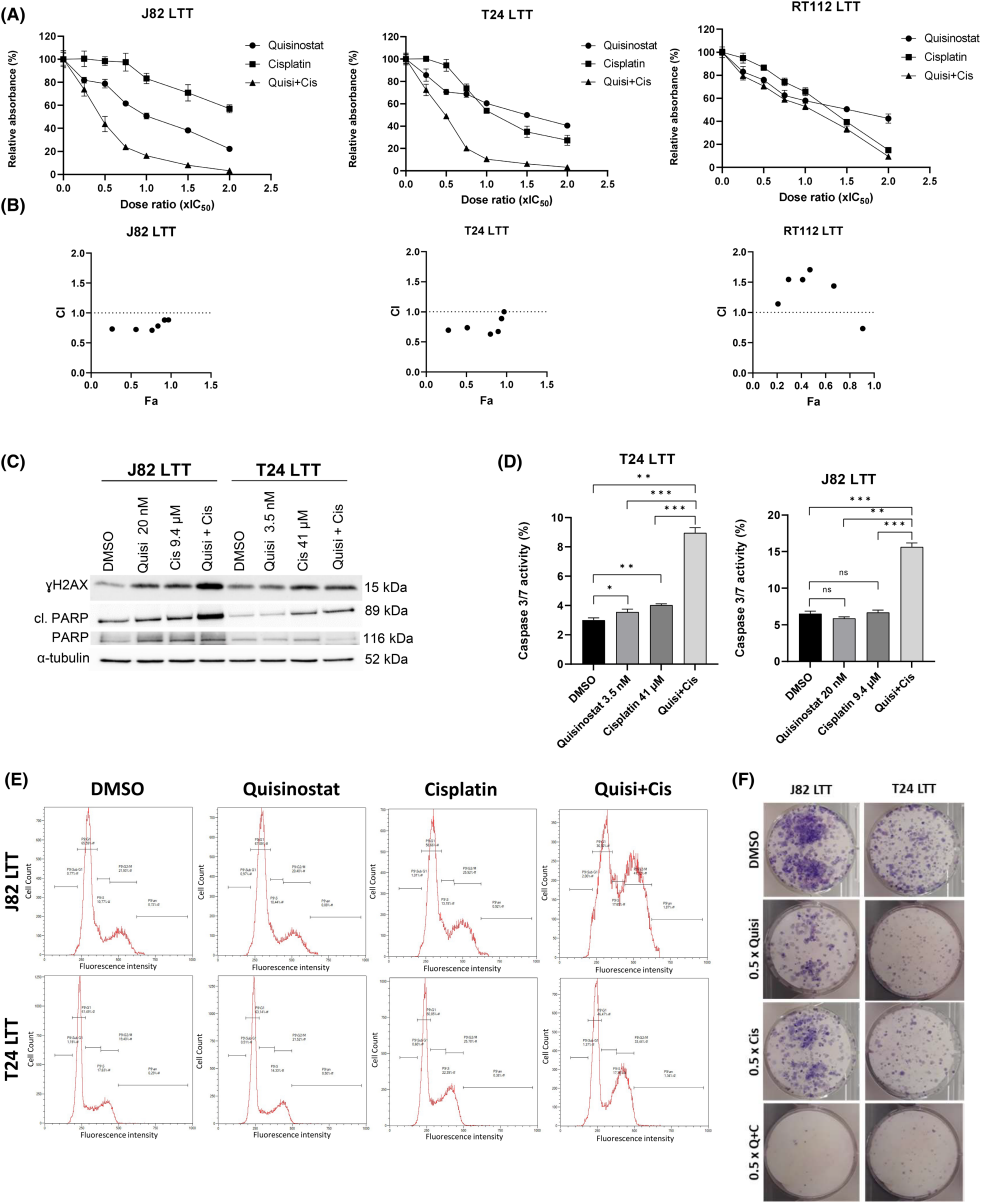
Quisinostat synergises with cisplatin in cells resistant to cisplatin. (A) MTT assays curves of the single and combined treatments with cisplatin and talazoparib 72 h post‐treatment (± standard deviation SD) in J82 LTT, T24 LTT, RT‐112 LTT (LTT, long term treated), *n* = 4; (B) Chou Talalay analysis of the combinations quisinostat/cisplatin, CI, combination index, Fa, fraction affected; CI = 1: additive effect; CI >1: antagonistic effect; CI <1: synergistic effect. (C) Western blots showing the apoptopic marker cleaved PARP (cl. PARP), total PARP and ɣH2AX in J82 LTT and T24 LTT treated with IC_25_ dosage of quisinostat (Quisi), cisplatin (Cis), and combined treatment (Quisi + Cis) after 72 h. α‐tubulin was used as loading control. (D) Caspase 3/7 activity assay in J82 LTT and T24 LTT upon treatment with 0.5 IC_50_ values of quisinostat (Quisi), cisplatin (Cis), and combined treatment (Quisi + Cis) after 72 h. The results were normalized to the amount of viable cells measured with CellTiterGlo and are presented as percentage ±SD (standard deviation). *p*‐values are indicated as follows: *p < 0.05; **p < 0.005; ***p < 0.0005; n.s., non‐significant; *n* = 3. (E) Histograms showing the cell cycle distribution of J82 LTT and T24 LTT following after 72 h treatment with cell line‐specific IC_25_ dosage of quisinostat, cisplatin, talazoparib as single agents and as combined treatment, DMSO was used as control. Results of a representative experiment are shown. (F) Giemsa staining of the colony formation assay in J82 LTT and T24 LTT, after treatment with cell line‐specific IC_25_ dosage of quisinostat (Quisi), cisplatin (Cis), as single agents and as combined treatment, DMSO was used as control. Results of a representative experiment are shown.

Combined treatment with cisplatin in J82 LTT and T24 LTT demonstrated that quisinostat also synergises with cisplatin in cisplatin‐resistant sublines already at low dose ratios, thus the dose of cisplatin can be reduced when quisinostat is added (Figure 5A,B). In the RT‐112 cisplatin‐resistant subline, which is particularly resistant to cisplatin (IC_50_ = 200 μM); however, no synergism was observed.

Further analyses at low dose ratio (0.5 × IC_50_) in J82 and T24 LTT revealed an increase in cleaved PARP, γH2AX (Figure 5C), and caspase 3/7 activity (Figure 5D) upon combined treatment. Cell cycle and long‐term proliferation were also affected with the combination quisinostat and cisplatin (Figure 5E,F).

**FIGURE 6:**
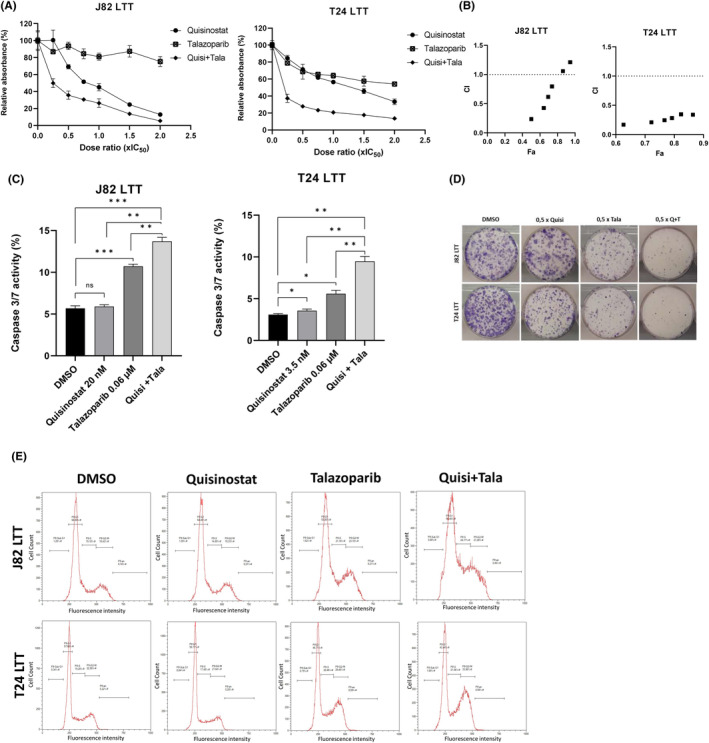
Quisinostat sensitizes J82 LTT and T24 LTT to the PARP inhibitor talazoparib. (A) MTT assays curves representing cell viability of the single and combined treatments with talazoparib 72 h post‐treatment in J82LTT and T24LTT (±Standard deviation SD); *n* = 4; (B) Chou Talalay analysis of the combinations quisinostat/talazoparib CI, combination index, Fa, fraction affected; CI = 1: additive effect; CI >1: antagonistic effect; CI <1: synergistic effect; (C) Caspase 3/7 activity assay in J82 LTT and T24 LTT upon treatment with quisinostat (IC_25_), 0.06 μM talazoparib, the combination of quisinostat and talazoparib. *p*‐values of *t*‐test results are indicated as follow: *p < 0.05; **p < 0.005; ***p < 0.0005; n.s., non‐significant; *n* = 3. (D) Giemsa staining of the colony formation assay for J82 LTT and T24 LTT, after treatment with cell line‐specific IC_25_ dosage of quisinostat (Quisi), talazoparib (Tala), as single agents and as combined treatment, DMSO was used as control. Results of a representative experiment are shown. (E) Histograms showing the cell cycle distribution of J82 LTT and T24 LTT following a 72 h treatment with cell line‐specific IC_25_ dosage of quisinostat, talazoparib as single agents and as combined treatment (Quisi + Tala), DMSO was used as control. Results of a representative experiment are shown.

Similar results were observed with the combination with talazoparib (Figure 6A–E). This combination was also highly synergistic in the cisplatin‐resistant setting, strongly inhibited cell growth and significantly induced apoptosis.

### 3.6 Effect of the combination on normal toxicity

**FIGURE 7:**
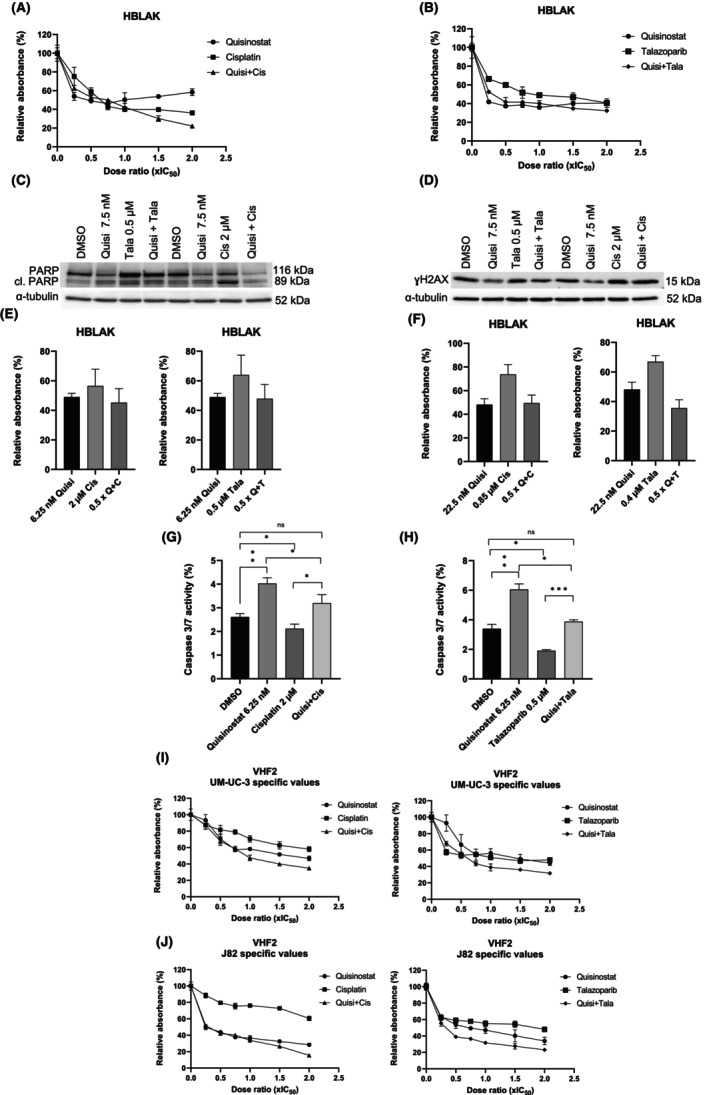
Effects of the combinations in normal cell lines. (A, B) MTT assays curves as a surrogate for cell viability of the single and combined treatments with cisplatin and talazoparib 72 h post‐treatment in HBLAK (± standard deviation SD) with HBLAK specific values (IC_50_ used for: quisinostat 15 nM, cisplatin 4 μM, talazoparib 1 μM); *n* = 4. (C) Western blots showing the apoptotic marker cleaved PARP and total PARP in HBLAK treated with IC_25_ dosage of quisinostat, cisplatin, talazoparib, and combined treatment after 72 h. α‐tubulin was used as loading control. (D) Western blots for ɣH2AX in HBLAK treated with IC_25_ dosage of quisinostat, cisplatin, talazoparib, and combined treatment after 72 h. α‐tubulin was used as loading control. The respective IC_50_ of the cell lines were used. Romi: romidepsin; Quisi: quisinostat. (E) Bar graph presenting the cell viability of HBLAK as relative absorbance (%) upon 72 h treatment (Quisi, quisinostat; Cis, cisplatin; Q + C, quisinostat + cisplatin) with 0.5 × IC_50_ values specific to UM‐UC‐3. (F) Bar graph presenting metabolic activity as a surrogate for cell viability of HBLAK as relative absorbance (%) upon 72 h treatment (Quisi, quisinostat; Cis, cisplatin; Q + C: quisinostat+cisplatin) with 0.5 × IC_50_ values specific to J82; *n* = 4. (G) Caspase 3/7 activity assay in HBLAK upon 72 h treatment with 6.25 nM quisinostat, 2 μM cisplatin and the combination of quisinostat and cisplatin. (H) Caspase 3/7 activity assay in HBLAK upon treatment with 6.25 nM quisinostat, 0.5 μM talazoparib, the combination of quisinostat and talazoparib. *p*‐values of *t*‐test results are indicated as follows: *p < 0.05; **p < 0.005; ***p < 0.0005; n.s., non‐significant; *n* = 3. (I, J) MTT assay curves of the single and combined treatments with cisplatin and talazoparib in VHF2 72 h post‐treatment with UM‐UC‐3 specific values (I) and J82 specific values (J); *n* = 4.

Both combinations were tested in normal cells (Figure 7). The strong synergism of combined treatment in UCC was not observed in HBLAK (Figure 7A,B). Further analyses in HBLAK indicated that there was no marked increase in cleaved PARP (Figure 7C) and γH2AX (Figure 7D). Additional analysis with UM‐UC‐3 and J82 specific values at 0.5 × IC_50_ revealed that there was neither a decrease in MTT absorbance values with the combinations (Figure 7E,F) nor a significant increase in caspase 3/7 activity (Figure 7G,H) in HBLAK. The combinations were also well tolerated by the fibroblasts VHF2 as no strong decrease in MTT absorbance was observed when UM‐UC‐3 and J82 specific values were used (Figure 7I,J). These results demonstrate that synergistic combinations were tolerated by normal cells without additional toxicity.

## 4 DISCUSSION

In this study, we evaluated the effect of the second generation HDACi quisinostat in a panel of UCC representing the heterogeneity of urothelial cancer as monotherapy and in combination with cisplatin or the PARP inhibitor talazoparib.

Previous studies in UCC^12^, ^13^ indicated that HDACi such as romidepsin and givinostat affected cell proliferation, proliferation ability, cell cycle progression with a G2/M arrest and apoptosis. Quisinostat is a second generation HDACi which was identified in a screen for class I HDACi with enhanced pharmacodynamic profile and was shown to inhibit cancer growth in different cancer types (breast, ovarian, lung, prostate, colon, brain).^18^

UM‐UC‐3 and J82 responded strongly to quisinostat with induction of caspase dependent apoptosis and accumulation of cells in G2/M. UCC rather accumulate in G2/M since most of commonly used UCC bear genetic alterations in cell cycle regulators.^13^ HDACi are indeed known to induce caspase‐dependent cell death.^41^, ^42^ The apoptosis array identified underlying proteins affected in UM‐UC‐3 and J82. The level of the apolipoprotein clusterin, which is associated with resistance to apoptosis following HDACi treatment^43^ was increased with quisinostat. The phosphorylated forms of p53 were all downregulated, suggesting that the signalling pathways leading to apoptosis do not function properly after quisinostat treatment. Of note, these cell lines present mutated version of TP53.^44^ The pro‐apoptopic proteins HTRA and SMAC were induced by quisinostat treatment, hinting that they mediate quisinostat induced‐apoptosis. Anti‐apoptotic proteins Survivin, Claspin and XIAP were reduced after 24 h treatment, but normalized after 72 h. In other cancer entities quisinostat had also been show to induce apoptosis by altering the balance of pro‐ and anti‐apoptotic proteins.^45^, ^46^

Interestingly, normal cells (uroepithelial HBLAK and dermal fibroblastsVHF2) tolerated high doses of quisinostat; which could be attributed to the genetic defects in cell cycle regulators present in UCC but not in normal cells, allowing the latter to arrest properly in cell ycle to repair the damages caused by the HDACi.^13^, ^15^, ^42^ Concurringly, staining of DNA double‐strand breaks revealed numerous stained foci and enlarged nuclei in treated UCC after 16 h that remained after 72 h. In contrast, in HBLAK increase in number of positively stained cells by quisinostat compared to DMSO was less prominent than in treated UCC. Also nuclear size seemed to be unaltered while overall cell size increased with higher dosage. DAPI staining appeared less intensive in treated HBLAK. Altogether, this suggests that some cells may acquire a senescence‐like phenotype after quisinostat treatment. Concurringly, levels of IL‐6 secretion, another senescence marker, was similarly increased 7 days after quisinostat treatment of HBLAK when compared with ionizing radiation.^47^ Likewise, senescent‐like cellular morphology looked comparable. Lastly, our experiments with a senolyticum after quisinostat pre‐treatment further support our hypothesis that HBLAK tolerate quisinostat better than UCC since they acquire senescence characteristics compared to UCC undergoing apoptosis.^48^

The treatment induced senescence‐like state of HBLAK appeared to be reversible after washout. Thus, even though HBLAK cells had increased β‐galactosidase activity, they obviously did not reach a permanently arrested state, which could be advantageous regarding side toxicity. To our knowledge this study is the first reporting about impact of quisinostat on senescence characteristics. We reported earlier about changes in β‐galactosidase activity by HDACi romidepsin, givinostat and vorinostat.^12^ However, effects induced by different HDACi may differ. While vorinostat is a pan‐HDAC inhibitor, romidepsin, givinostat and quisinostat target mainly class I HDACs. Still, effects of class I HDACi may also differ. While romidepsin mainly targets HDAC1 and 2, quisinostat is most potent to target HDAC1. Concurring with differences in their isoenzyme specificity, the difference between romidepsin and quisinostat can also be explained by their effect on gene expression, which was shown to differ in a recent study in gliomas.^49^ We also observed overall differences in transcriptome of romidepsin and quisinostat treated UC cell lines. One important difference is their impact on DNA damage response signalling.

Generally, senescence may be one option for cellular response to induced DNA damage.^48^ Since genetic alterations in cell cycle regulators like p53, RB, and p16 that are related to senescence induction are very common in UC cell lines,^7^, ^44^ these may be the reason why quisinostat UC cells acquired less prominent characteristics of senescence.

Induction of DNA damage by quisinostat in cancer cells suggested to test combinations with the DNA damaging compound cisplatin or a PARP inhibitor affecting DNA damage repair. Other HDACi have shown promising results in combination with cytotoxic agents targeting DNA (cisplatin, topoisomerase inhibitor) to enhance cytotoxicity against cancer cells.^50^, ^51^, ^52^, ^53^ Previous preclinical studies demonstrated that quisinostat was an appropriate combination partner for CDKi in melanoma, doxorubicin in breast cancer, cisplatin in lung cancer, and bortezomib in synovial sarcoma.^27^, ^54^, ^55^, ^56^

Here, we report for UCC that quisinostat could be combined with cisplatin or with the PARP inhibitor talazoparib to amplify its cytotoxic effect, resulting in a higher proportion of cell death with reduced dosages. Synergism was even observed in cisplatin‐resistant LTTs, suggesting that these combinations could also be interesting options to tackle acquired resistance. Enhanced effect of HDACi and cisplatin has been attributed to decompaction of chromatin induced by HDACi, enabling it to be accessible for more cisplatin‐induced damages.^57^ Additionally, we observed that quisinostat led to an increase in the DNA double strand break marker γH2AX, suggesting that it could induce DNA damage adding to the amount of DNA damage when combined with cisplatin.^16^

The PARP inhibitor talazoparib functions by trapping PARP1 to the DNA, preventing PARylation, DNA repair and replication.^58^ We observed that the combination of quisinostat and talazoparib was synergistic at low dose ratio, accompanied by disturbance of cell cycle, increased caspase‐dependent apoptosis and γH2AX. Other studies demonstrated that addition of the PARPi olaparib to the pan‐HDACi SAHA improved the efficiency of the single treatment, leading to impeded cell cycle and a downregulation of key proteins involved in DNA damage repair (*BRCA1, RAD51*).

In conclusion, our promising results recommend combination of quisinostat with cisplatin or talazoparib as new treatment options for UC. These combinations were also efficient in cisplatin‐resistant cell lines and may thus have a potential to tackle chemoresistance in urothelial cancer. Application of the combined treatment to normal cells suggested low normal toxicity.

## AUTHOR CONTRIBUTIONS

**Sarah Meneceur:** Conceptualization (equal); data curation (equal); formal analysis (equal); investigation (equal); methodology (equal); software (equal); validation (equal); visualization (equal); writing – original draft (equal); writing – review and editing (equal). **Caroline E. De Vos:** Formal analysis (equal); investigation (equal); writing – review and editing (equal). **Patrick Petzsch:** Data curation (equal); formal analysis (equal); investigation (equal); methodology (equal); software (equal); writing – review and editing (equal). **Karl Köhrer:** Methodology (equal); resources (equal); writing – review and editing (equal). **Günter Niegisch:** Conceptualization (equal); funding acquisition (equal); project administration (equal); resources (equal); supervision (equal); writing – original draft (equal); writing – review and editing (equal). **Michèle J. Hoffmann:** Conceptualization (equal); data curation (equal); formal analysis (equal); funding acquisition (equal); investigation (equal); methodology (equal); project administration (equal); software (equal); supervision (equal); visualization (equal); writing – original draft (equal); writing – review and editing (equal).

## FUNDING INFORMATION

This research was funded by the German research council DFG by a project grant to M.J.H. and G.N., grant no. HO 6448/NI 1398.

## CONFLICT OF INTEREST STATEMENT

The authors declare no conflict of interest. The funders had no role in the design of the study; in the collection, analyses, or interpretation of data; in the writing of the manuscript, or in the decision to publish the results.

## Supporting information


Figure S1.

Figure S2.

Figure S3.

Figure S4.

Figure S5.

Figure S6.

Figure S7.

## Data Availability

The data presented in this study is comprehensively shown in this article and its supplementary files. The underlying unprocessed raw data is available upon request.
