# Supplementary figures and images for: New synergistic combination therapy approaches with HDAC inhibitor quisinostat, cisplatin or PARP inhibitor talazoparib for urothelial carcinoma
Source: J Cell Mol Med. 2024 May 2;28(9):e18342. doi: 10.1111/jcmm.18342 (PMC11063726; doi:10.1111/jcmm.18342)

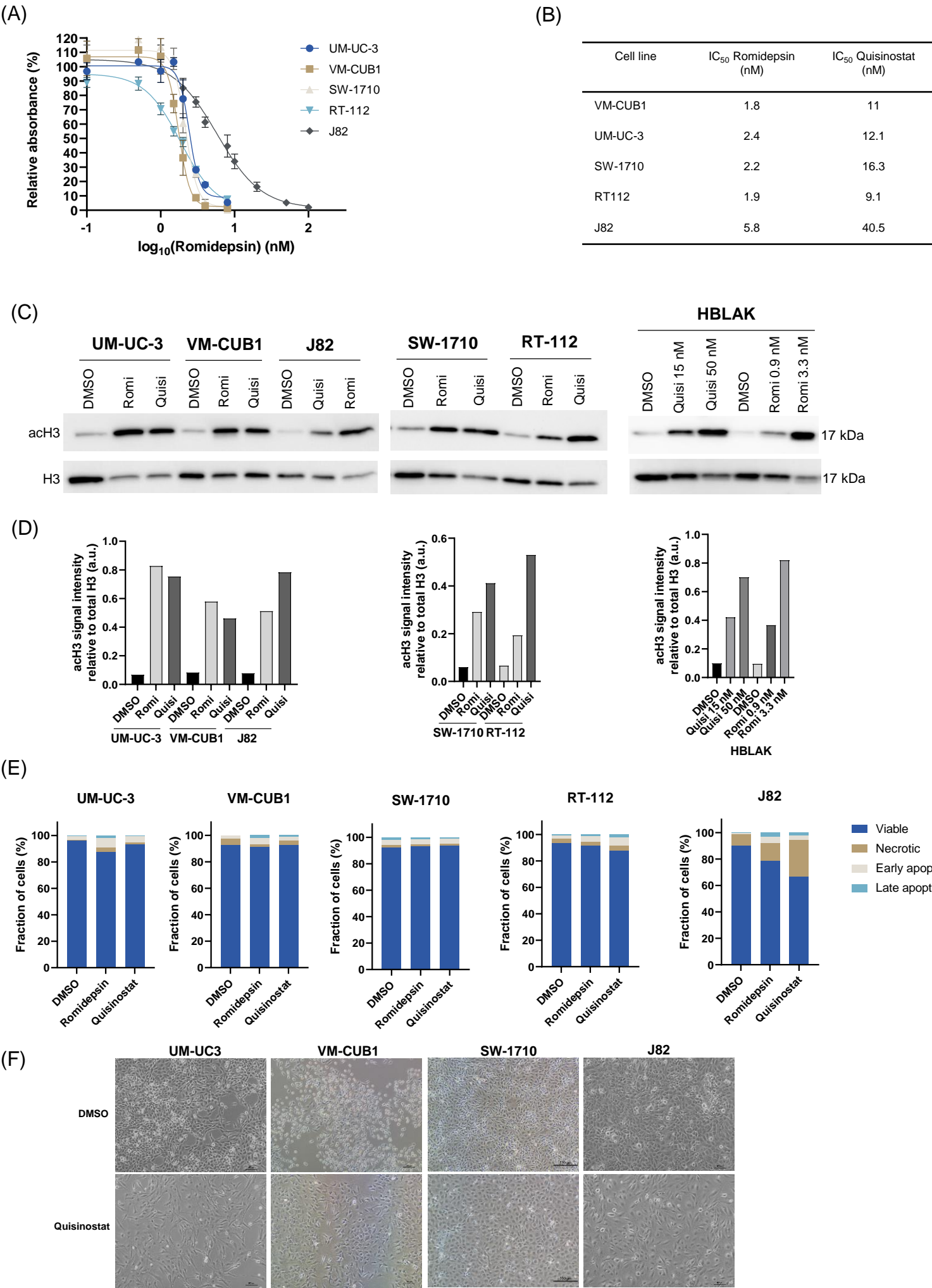

Supplementary Fig.S1

Supplement: Supplementary file 1 — Figure S1. [file JCMM-28-e18342-s004.pdf]

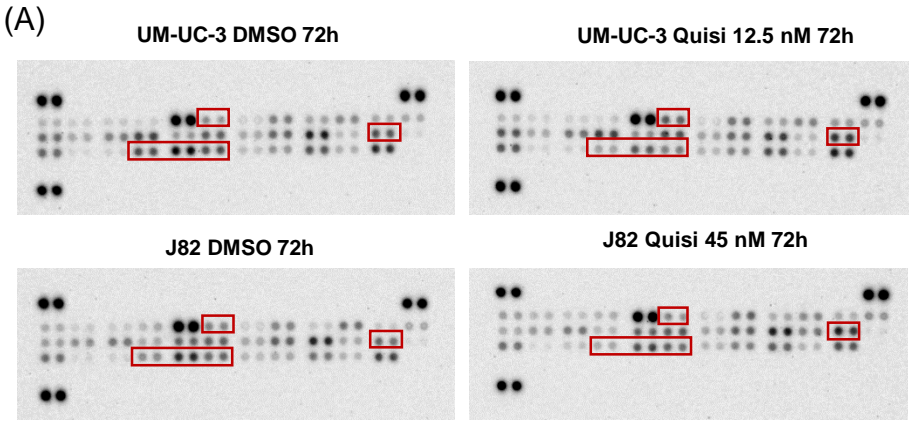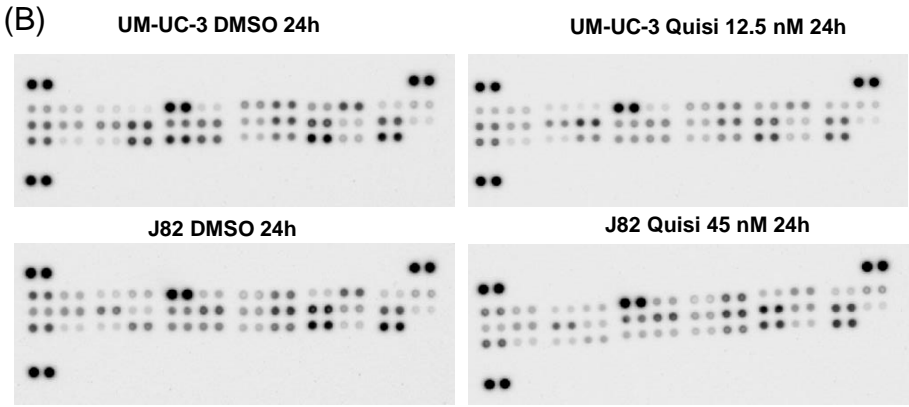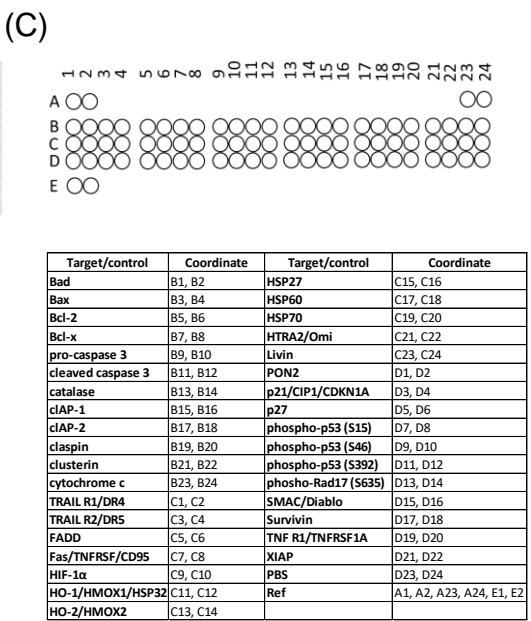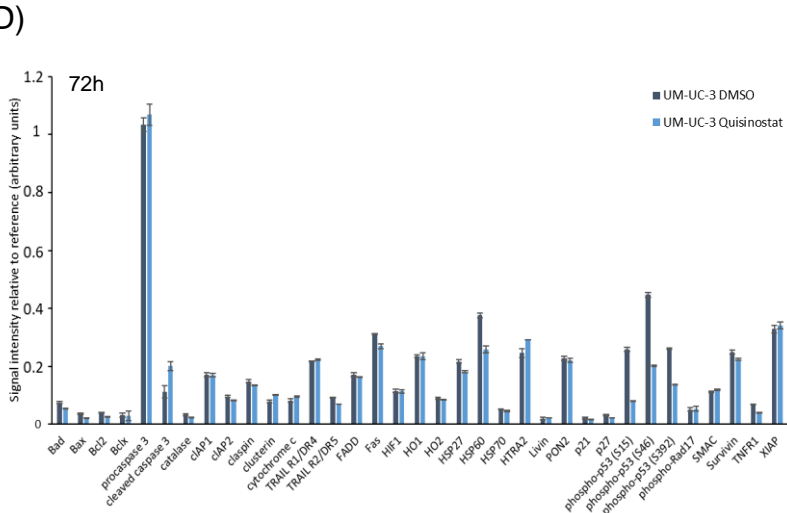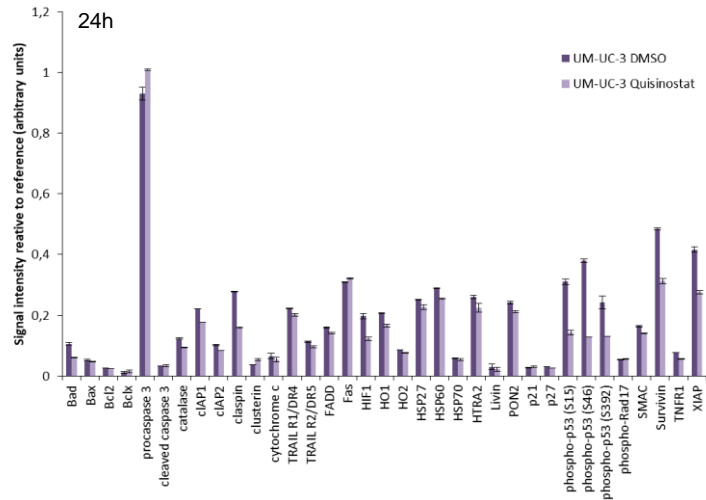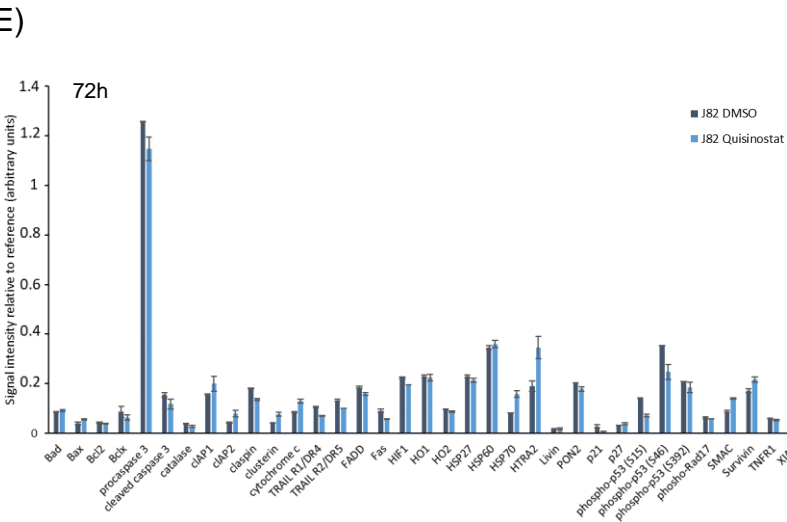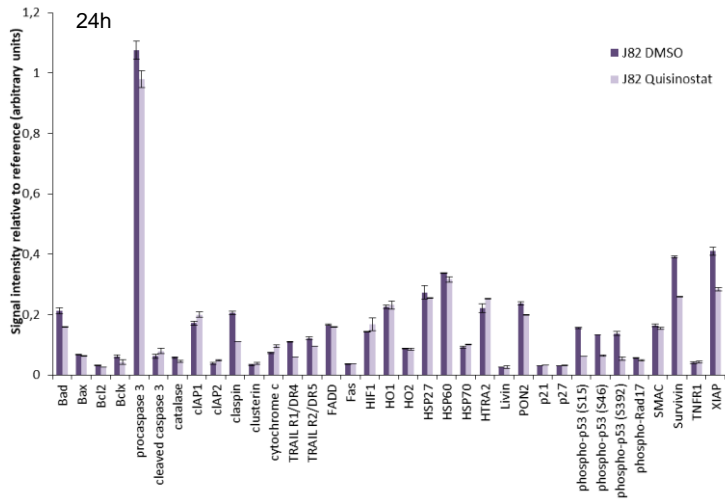

Supplementary Fig. S2

Supplement: Supplementary file 2 — Figure S2. [file JCMM-28-e18342-s005.pdf]

## (A) HBLAK ICC

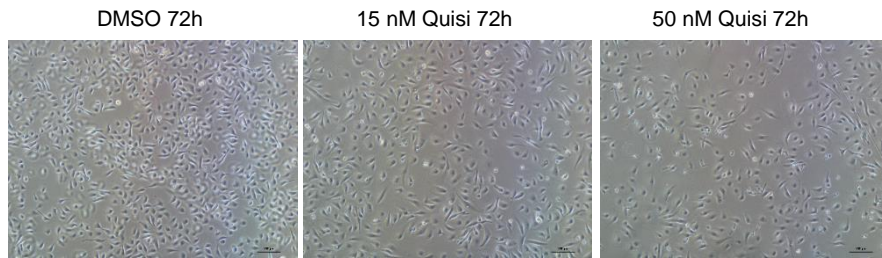

## (B) HBLAK FACS senescence

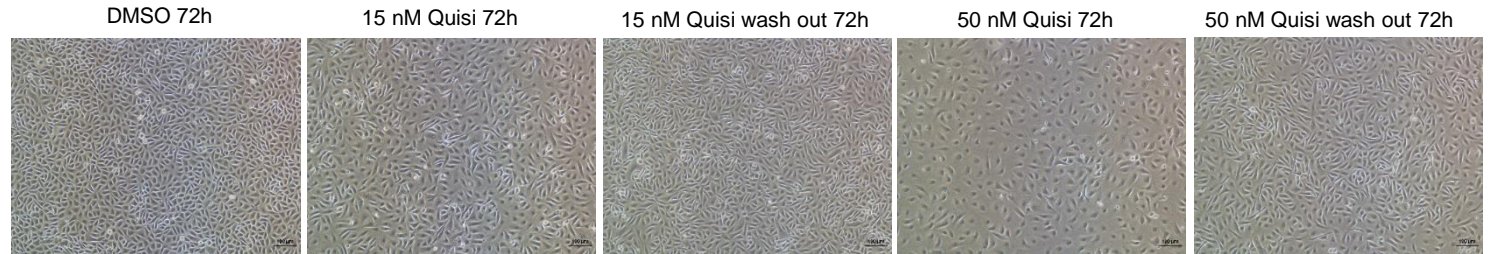

## (C)

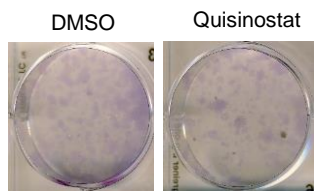

## (D)

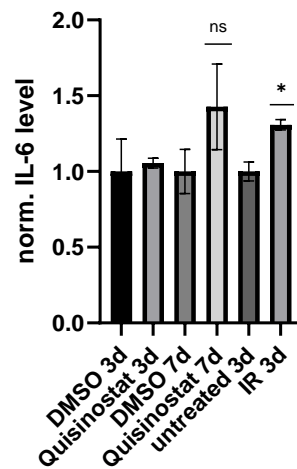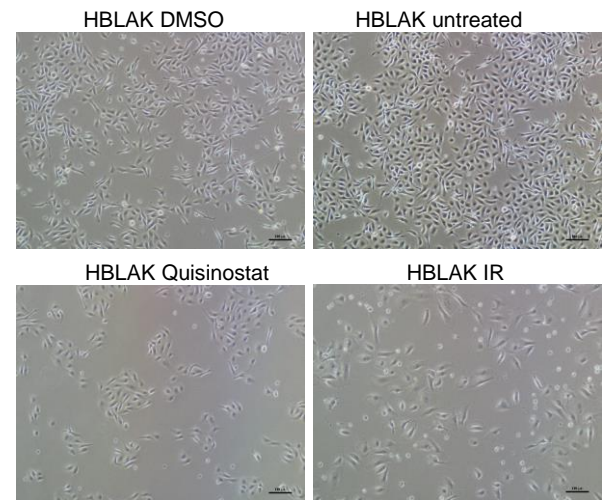

## (E)

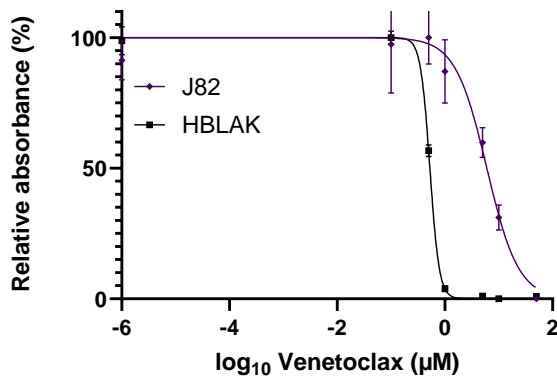

## (F)

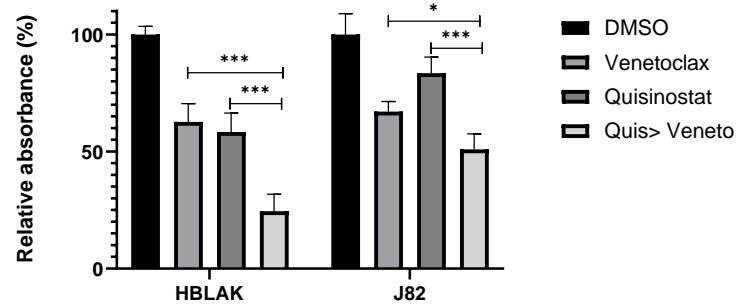

Supplement: Supplementary file 3 — Figure S3. [file JCMM-28-e18342-s003.pdf]

(A)

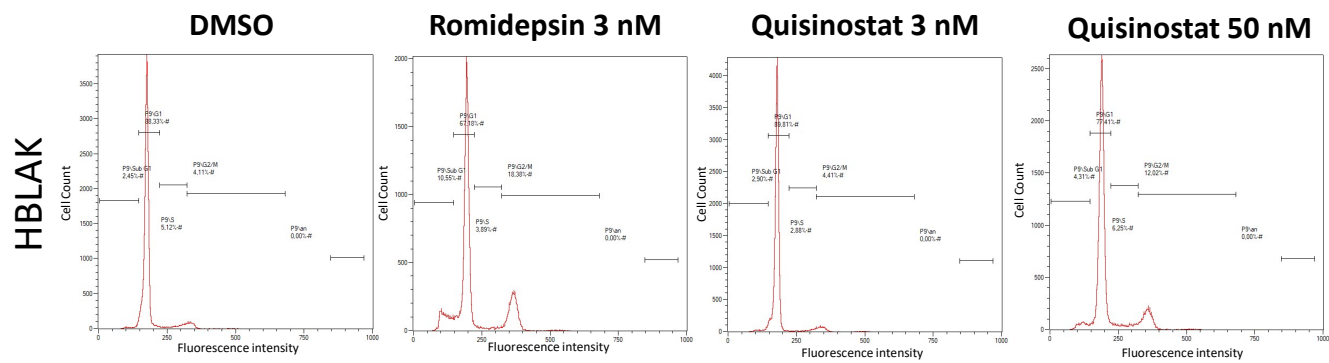

(B)

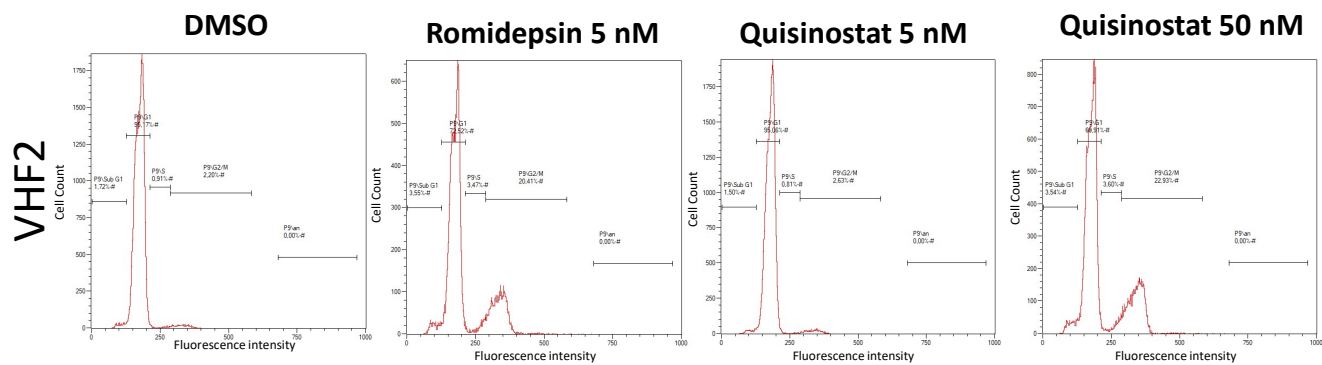

Supplement: Supplementary file 4 — Figure S4. [file JCMM-28-e18342-s007.pdf]

(A)

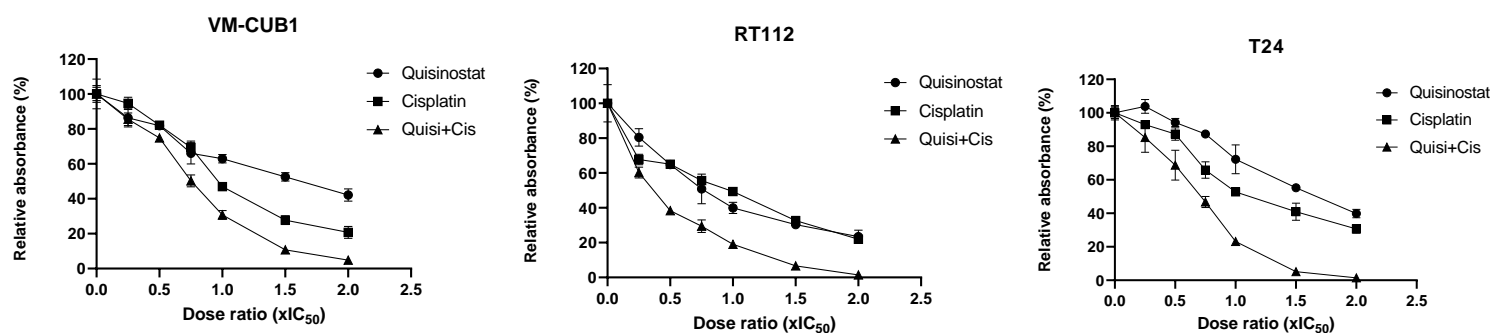

(B)

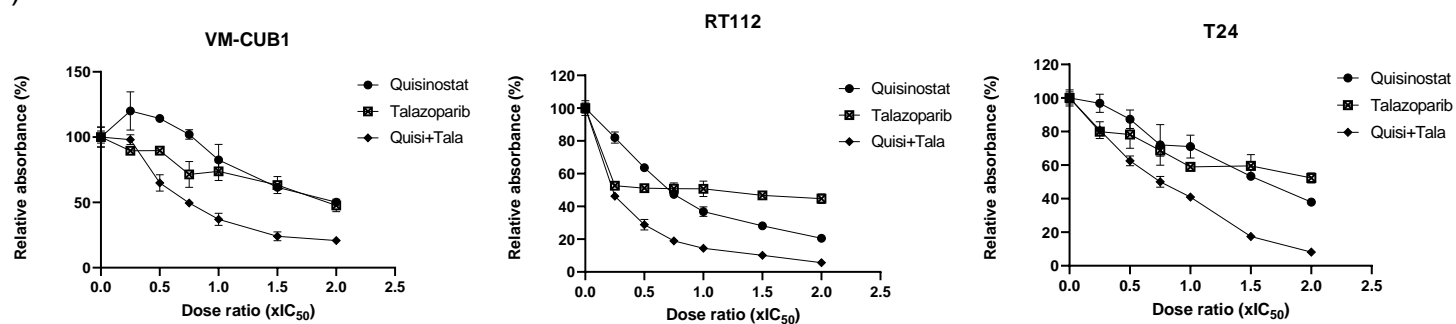

(C)

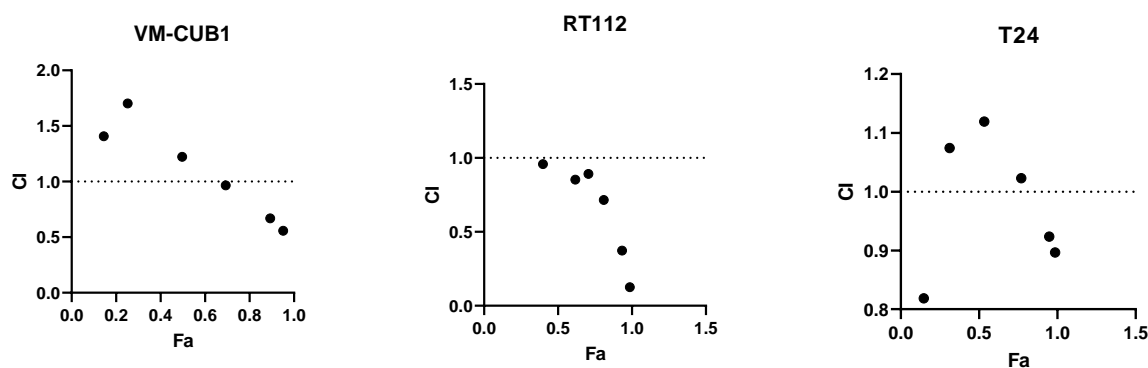

(D)

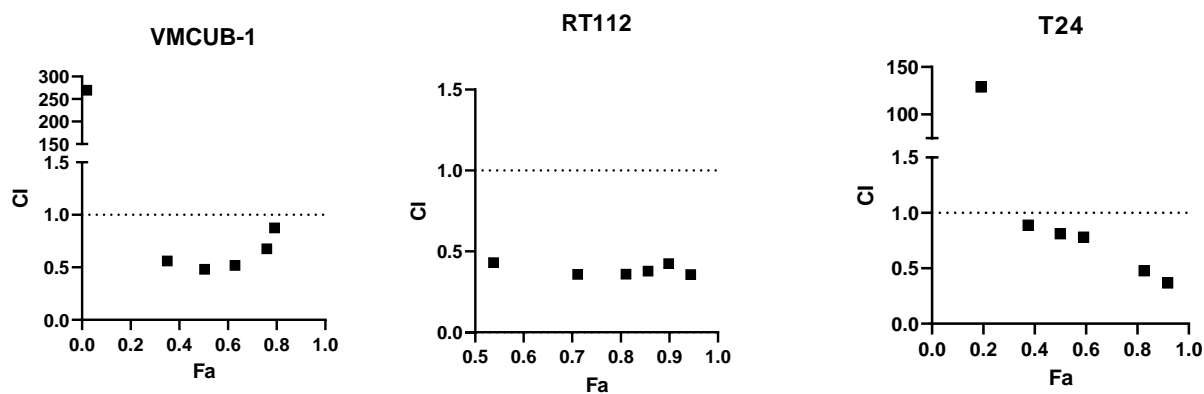

Supplement: Supplementary file 5 — Figure S5. [file JCMM-28-e18342-s002.pdf]

(A)

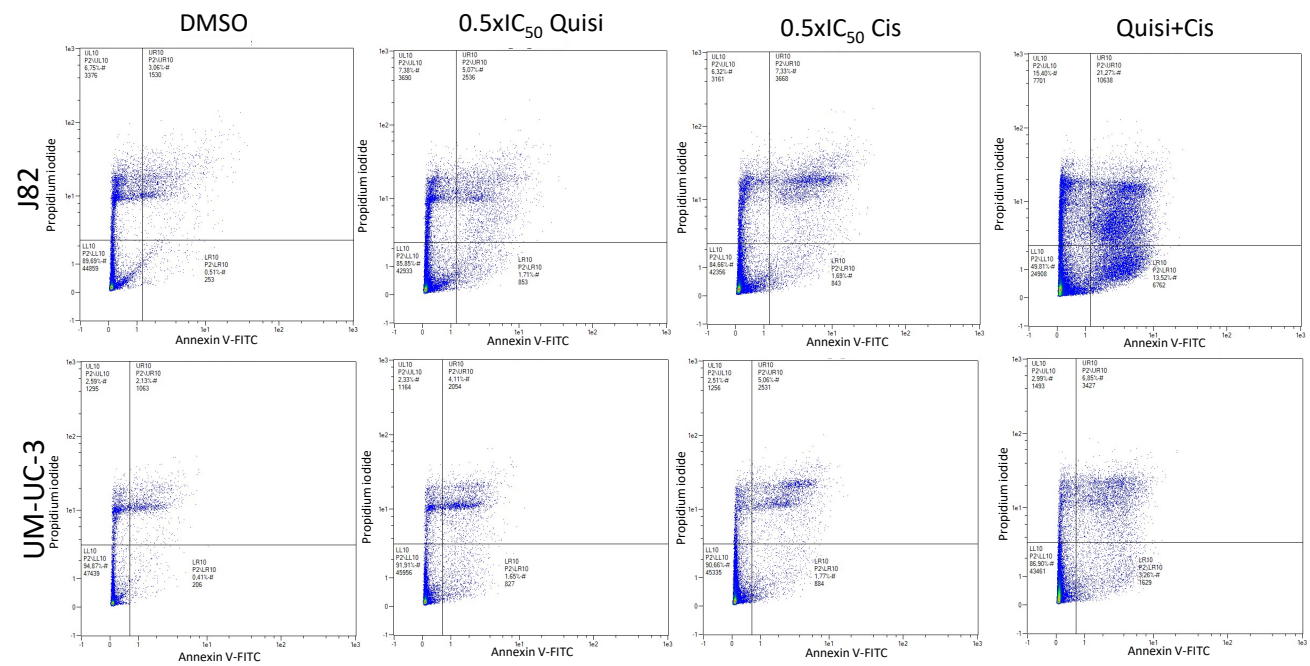

(B)

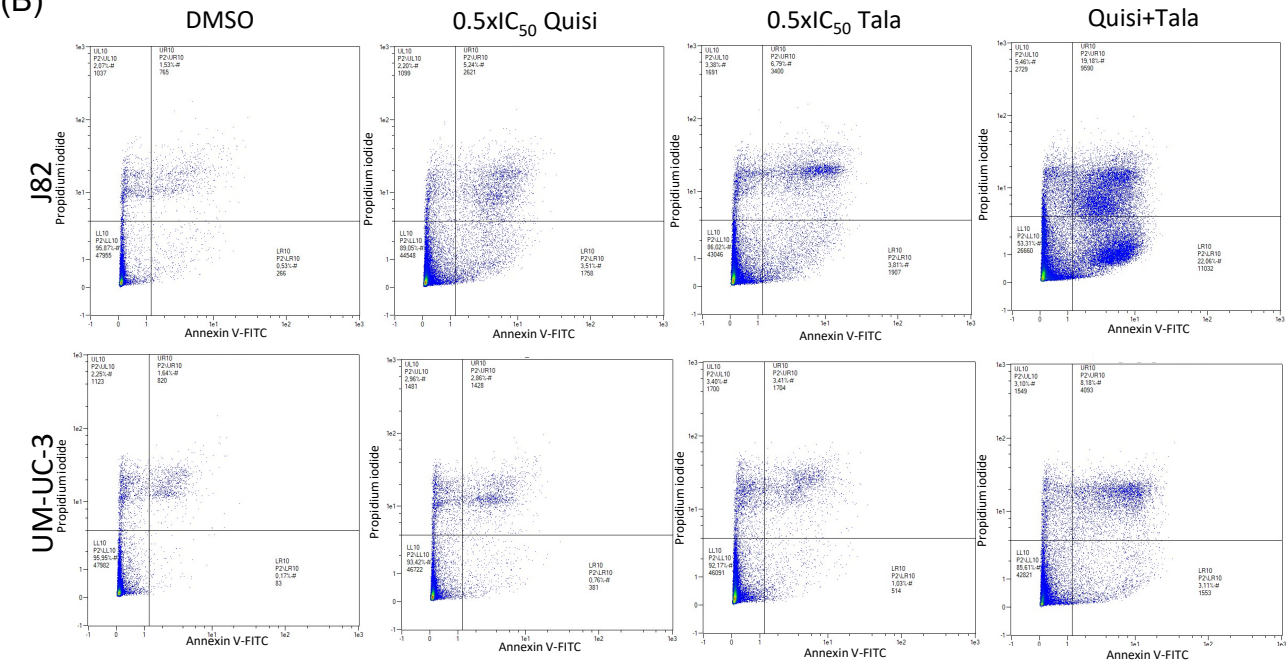

Supplement: Supplementary file 6 — Figure S6. [file JCMM-28-e18342-s006.pdf]

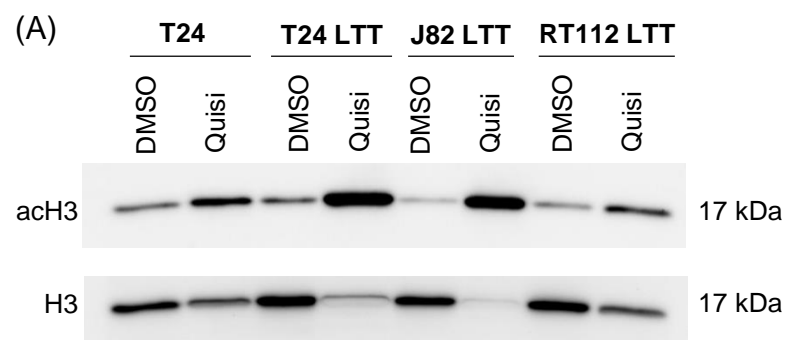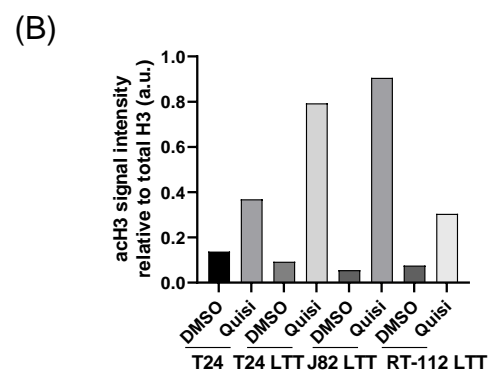

Supplement: Supplementary file 7 — Figure S7. [file JCMM-28-e18342-s001.pdf]
